# Erythropoiesis-Stimulating Agent Protects Against Kidney Fibrosis by Inhibiting G2/M Cell Cycle Arrest

**DOI:** 10.3390/cells14211662

**Published:** 2025-10-23

**Authors:** Donghwan Oh, Jong Hyun Jhee, Soo Hyun Kim, Tae Yeon Kim, Hyo Jeong Kim, Wooram Bae, Hoon Young Choi, Hyeong Cheon Park

**Affiliations:** 1 Division of Nephrology, Department of Internal Medicine, Gangnam Severance Hospital, Yonsei University College of Medicine, Seoul 03722, Republic of Korea; donghwan23@gmail.com (D.O.); jjhlove77@yuhs.ac (J.H.J.); bwr@yuhs.ac (W.B.); hychoidr@yuhs.ac (H.Y.C.); 2 Severance Institute for Vascular and Metabolic Research, Yonsei University College of Medicine, Seoul 03722, Republic of Korea

**Keywords:** chronic kidney disease, kidney injury, renal fibrosis, cell cycle arrest, erythropoiesis stimulating agent

## Abstract

**Background:** G2/M cell cycle arrest of proximal tubular epithelial cells following acute kidney injury results in maladaptive repair and promotes chronic kidney disease. We investigated whether erythropoiesis-stimulating agents (ESA) regulate G2/M arrest and mitigate kidney fibrosis. **Methods:** Human kidney 2 (HK-2) cells were stimulated with TGF-β or paclitaxel, treated with darbepoetin alfa (DARB) at 0.5 ug/mL or 5 ug/mL, and cell cycles were analyzed using flow cytometry. In vivo experiments involved intraperitoneal administration of DARB (0.5 or 5 ug/kg) to the unilateral ureteral obstruction (UUO) mouse model on post-operative days three and seven. Kidney fibrosis and cell cycle regulatory proteins were analyzed using immunohistochemistry, RT-PCR, and immunoblotting. The effect of DARB on kidney fibrosis was compared with that of a p53 inhibitor. **Results:** In HK-2 cells treated with TGF-β or paclitaxel, G2/M cell cycle regulatory proteins were upregulated; however, this effect was reversed by DARB treatment. Immunostaining for p53 and Ki-67 indicated that the proliferative and fibrotic activities observed in TGF-β-treated HK-2 cells were mitigated by DARB treatment. Histological analysis of UUO mice using F4/80 staining and TUNEL assay showed that DARB treatment reduced inflammatory cell infiltration and apoptotic cell accumulation. Additionally, fibrotic changes assessed by Masson’s trichrome, Sirius red, and PAS staining confirmed the antifibrotic effects of DARB treatment in UUO mice, independent of changes in hemoglobin levels, suggesting a mechanism distinct from its hematopoietic effects. DARB reduced fibrosis-related markers by suppressing G2/M cell cycle regulatory markers and inhibited the JNK and p38-MAPK signaling pathways, which play key roles in kidney fibrosis in TGF-β-treated HK-2 cells and UUO mice. Finally, DARB treatment demonstrated an anti-fibrotic effect in HK-2 cells stimulated with TGF-β or paclitaxel, comparable to that of a p53 inhibitor. **Conclusions:** DARB treatment decreased G2/M cell phase arrest and attenuated kidney fibrosis, suggesting a new renoprotective mechanism for ESA.

## 1. Introduction

Kidney fibrosis is a key feature in the development of chronic kidney disease (CKD) [1]. Pathological hallmarks of fibrotic changes in CKD include interstitial fibroblast proliferation, persistent extracellular matrix (ECM) deposition, epithelial-to-mesenchymal transition (EMT) of tubular epithelial cells, and interstitial tubular atrophy [2,3,4,5,6,7,8]. Acute kidney injury (AKI), prevalent among hospitalized patients and associated with poor prognosis upon recurrence, often transitions to CKD because of incomplete repair [9,10,11,12]. Residual kidney damage sustains inflammation in the tubulointerstitial parenchyma [13,14], driving fibroblast proliferation and ECM deposition, thereby exacerbating tubulointerstitial fibrosis.

Emerging evidence suggests that epithelial cell cycle arrest is a key driver of kidney fibrosis post-AKI [7,15,16,17]. Although G1 phase arrest serves as an ideal and early biomarker for predicting AKI, G2/M phase arrest in proximal tubular epithelial cells is closely associated with the progression from severe AKI to CKD [7,15]. Erythropoietin (EPO), primarily recognized for its hematopoietic function [3], also exhibits potent anti-inflammatory and anti-apoptotic properties and protects against kidney fibrosis. Notably, recombinant human EPO has demonstrated efficacy in attenuating kidney fibrosis in a unilateral ureteral obstruction (UUO) rat model by suppressing TGF-β-induced EMT and reducing TNF-α expression and inflammatory cell infiltration [18,19]. Beyond its hematopoietic effects, EPO has been implicated in modulating cell cycle dynamics. EPO treatment enhances the G1-to-S transition in erythroblasts by inhibiting regulatory factors [20]. However, the potential relationship between EPO’s non-hematopoietic effects on cell cycle arrest and its protective role against kidney fibrosis remains poorly understood.

In this study, we hypothesized that EPO modulates G2/M cell cycle arrest in kidney tubular epithelial cells, thereby mitigating kidney fibrosis. We investigated the effects of an erythropoiesis-stimulating agent (ESA), darbepoetin alfa (DARB) on kidney fibrosis progression, focusing on its role in cell cycle modulation.

## 2. Materials and Methods

### 2.1. Human Kidney 2 (HK-2) Cell Culture and DARB Treatment

Human kidney (HK-2) cells (cat. no. CRL-2190, American Type Culture Collection, Manassas, VA, USA) were cultured in Dulbecco’s modified Eagle’s medium (Thermo Fisher Scientific, Waltham, MA, USA) until the cell area was 80% confluent at 33 °C. HK-2 cells were seeded onto culture plates for 48 h in a complete medium containing 10% fetal bovine serum (FBS). The cells were kept in serum-free medium for 24 h and placed in the 1% FBS-added medium for 48 h or treated with recombinant human TGF-β (5 ng/mL, R&D Systems, Minneapolis, MN, USA) or paclitaxel (10 nM, Sigma-Aldrich, St. Louis, MO, USA) for 48 h. The medium was changed and the cells that were treated with TGF-β or paclitaxel were continuously treated with or without DARB (darbepoetin alfa, Nesbell^®^, Chong Kun Dang Pharmaceutical Corp, Seoul, Republic of Korea) at low (0.5 ug/mL) or high (5 ug/mL) doses, along with a p53 inhibitor (pifithrin-α; 20 uM; cat. No. P4359, Sigma-Aldrich, St. Louis, MO, USA). The doses of DARB used in this study were chosen based on prior literature and preliminary in vitro data, ensuring minimal cytotoxicity while maintaining biological efficacy in kidney cells [21,22]. For the EPO receptor (EPOR) blockade experiment, HK-2 cells were seeded in 6-well plates and pretreated with an EPOR-blocking peptide (MyBioSource, San Diego, CA, USA, 5 μM) for 1 h, followed by stimulation with TGF-β1 (5 ng/mL) and DARB (0.5 or 5 μg/mL) for 48 h.

### 2.2. Animal Experiments

All experiments in the present study were approved by the Institutional Animal Care and Use Committee (IACUC, no 2021-0053) of the Yonsei University Health System (Seoul, South Korea) and conducted in accordance with its guidelines. Adult CD1 mice were purchased from Orient Bio, Inc. (Seongnam, Republic of Korea). All animal study protocols were designed in accordance with the Guidelines for the Use of Laboratory Animals. The animals were housed under temperature-controlled conditions with a 12-h light/dark cycle and provided water and food ad libitum. UUO was performed following this protocol: Briefly, CD1 mice were anesthetized using a combination of isoflurane and oxygen and placed on a heating pad (Jeung Do Bio & Plan Co, Seoul, Republic of Korea) to maintain their body temperature at 37 °C. The left ureter was exposed using a flank incision and ligated with 3-0 silk at two points immediately below the lower pole of the left kidney, followed by suturing of the peritoneal membrane and skin. Following surgery, the mice were randomly divided into four groups and administered vehicle (150 uL normal saline), low-dose DARB (0.5 ug/mL), or high-dose DARB (5 ug/mL) intraperitoneally (N = 5–6 per group). Blood pressure was monitored using tail cuffs. All mice were sacrificed three or seven days after UUO surgery to harvest the unilaterally obstructed kidneys for tissue collection. During mouse sacrifice, blood was collected through cardiac puncture, and hemoglobin levels were measured. Half of the kidney tissue was fixed in 4% paraformaldehyde for subsequent histological and immunohistochemical analysis, and the remaining half was frozen at −70 °C for protein isolation.

### 2.3. Analysis of Gene Expression by Quantitative RT-PCR

Total RNA was isolated from HK-2 cells and renal cortex using the TRIzol reagent (Invitrogen, Life Technologies, CA, USA). Reverse transcription was performed using 2 ug of total RNA with a High-Capacity cDNA Reverse Transcription Kit (Applied Biosystems, Bedford, MA, USA). The cDNA was amplified in the ABI 7500 sequence detection system (Applied Biosystems, Bedford, MA, USA) using Power SYBR^®^ Green PCR Master Mix (Applied Biosystems, Bedford, MA, USA). The cycling conditions were as follows: 40 cycles of 95 °C for 5 s, 58 °C for 10 s, and 72 °C for 20 s. Primer/probes (Macrogen, Seoul, Republic of Korea) were used to amplify *p53*, *p21*, *TGFB*, *cyclin B1*, *cyclin D1*, *CTGF*, *MCP-1*, *COL1A1*, and fibronectin as well as *GAPDH* (glyceraldehyde-3-phosphate dehydrogenase), which was used as the normalization control [23,24,25]. The expression of *GAPDH* remained stable across all experimental conditions. The PCR primer sequences are listed in Supplementary Table S1. Target gene expression was normalized to that of *GAPDH*. Relative quantification of gene expression was performed using the comparative CT method (^ΔΔ^CT method) using the StepOne Software version 2.2.2.

### 2.4. Histopathological Evaluation, Immunohistochemical Analysis, and Immunofluorescence Analysis

To analyze kidney fibrosis and cell cycle proteins, kidney sections were stained with Periodic acid-Schiff (PAS), Masson’s trichrome (TRC), and Sirius red (SR) stains. The stained sections were examined for TGF-β (cat. no. ab92486; Abcam, Cambridge, MA, USA), α-SMA (cat. no. MAB1420; R&D Systems, Minneapolis, MN, USA), p53 (cat. no. sc-126; 1:1000; Santa Cruz, Dallas, TX, USA), and p-HH3 (cat. no. 9701; Cell Signaling Technology) using light microscopy. Tubular injury assessed by PAS staining was scored at six levels based on the percentage of tubular dilation, epithelial desquamation, and loss of brush border in 10 randomly chosen non-overlapping fields at 400× magnification as follows: 0, none; 0.5, <10%; 1, 10–25%; 2, 25–50%; 3, 50–75%; and 4, >75%. The TRC-, SR-, anti-α-SMA-, and TGF-β-positive staining areas were evaluated relative to the unit area and expressed as a percentage per unit area using the MetaMorph microscopy image analysis software version 7.1 (Molecular Devices, Sunnyvale, CA, USA). Macrophage immunohistochemistry was performed using an anti-F4/80 antibody (cat. no. sc-52664; Abcam, Cambridge, MA, USA), and the F4/80-positive cell area was quantified as the number of cells per high-power field. The p53- and p-HH3-stained cells were also quantified per high-power field. TUNEL-positive cells were observed under a fluorescence microscope. Blinded microscopy assessment was performed using 20 randomly selected fields from each slide section examined at 400× magnification. Immunofluorescence staining of kidney sections was performed when the cells reached 70–80% confluency. The culture medium was removed, and the cells were rinsed once with 500 uL phosphate-buffered saline (PBS) for 5 min. After being fixed with 4% formaldehyde for 10 min, the HK-2 cells were permeabilized with 0.3% Triton X-100 (Sigma-Aldrich, St. Louis, MO, USA) for 15 min at 25–30 °C temperature. The cells were then incubated overnight at 4 °C with primary antibodies against Ki-67 (cat. no. ab15580; Abcam, Cambridge, MA, USA), p53, α-SMA, collagen (cat. no. sc-59772; Santa Cruz, Dallas, TX, USA), and fibronectin (cat. no. ab2413; Abcam, Cambridge, MA, USA). The next day, the cells were washed three times with PBS and incubated with goat anti-rabbit secondary antibody (Abcam, Cambridge, MA, USA) at 37 °C for 1 h. Following incubation with the secondary antibody, the cells were stained with DAPI (Abcam, Cambridge, MA, USA) for 5 min. After a final wash with PBS, images of the cells were captured using a laser-scanning confocal microscope (Leica, Buffalo Grove, IL, USA).

### 2.5. Immunoblotting of Renal Fibrosis Marker and Cell Cycle Proteins

For Western blotting and in vitro cell experiments, total cell protein was extracted from HK-2 cells using radioimmunoprecipitation assay (RIPA) buffer (Thermo Fisher Scientific, Waltham, MA, USA) and a Protein Extraction Kit (Bio-Rad, Hercules, CA, USA), following the manufacturer’s instructions. Protein concentrations were determined by the Bradford protein assay (Bio-Rad, 500-0006, Hercules, CA, USA). Protein samples (20 ug) were separated using 12% sodium dodecyl sulfate-polyacrylamide gel electrophoresis (SDS-PAGE) and transferred to polyvinylidene fluoride membranes (Millipore, Bedford, MA, USA). The membranes were first blocked with 5% skimmed milk for 1 h at 25–30 °C temperature, and incubated with antibodies. Detailed information regarding the primary and secondary antibodies used is provided in the Supplementary Methods. The bands were visualized using a chemiluminescent reagent (SuperSignal West Pico Luminol/Enhancer solution; Thermo Fisher Scientific, Waltham, MA, USA) and Agfa medical X-ray film. In the UUO mouse model, mouse kidney tissues were lysed in 300 uL of cell lysis buffer containing 2% sodium dodecyl sulfate, 62.5 mM Tris, pH 6.8, 0.01% bromophenol blue, 1.43 mM mercaptoethanol, 0.1% glycerol, and a protease inhibitor cocktail (Thermo Fisher Scientific, MA, USA). These kidney tissue lysates were resolved by SDS-PAGE, transferred onto polyvinylidene fluoride membranes, blocked with 5% skimmed milk, and incubated with primary antibodies similar to those used in the HK-2 cell experiment. The membranes were then washed thrice with 1% PBS containing Tween-20 for 5 min, incubated with horseradish peroxidase-conjugated secondary antibodies, and washed again using the same procedure. Equal amounts of protein, determined through the Bradford method, were loaded in each lane and normalized by β-actin [26,27]. The expression of β-actin did not vary between groups, ensuring reliable normalization of target protein. Target proteins used in previous HK-2 cell experiments were also visualized.

### 2.6. Flow Cytometry and Fluorescence-Activated Cell Sorting (FACS)

The cell cycle of HK-2 cells was assessed using flow cytometry with a BD FACSCanto II flow cytometer (BD Biosciences, San Jose, CA, USA). Briefly, HK-2 cells were trypsinized, fixed with 70% ice-cold ethanol, and stained with propidium iodide solution (Sigma-Aldrich, St. Louis, MO, USA). Cell cycle analysis was performed using the FlowJo software version 10.4.

### 2.7. Statistical Analysis

Quantitative analysis was performed using Western blotting and RT-PCR. Data were expressed as the mean ± standard deviation and statistically analyzed using the SPSS software version 25.0 (IBM-SPSS Inc., Armonk, NY, USA). Comparisons between groups were made using a one-way analysis of variance (ANOVA), followed by the Student–Newman–Keuls test. The multiple variance test was applied only when a significant difference was identified through one-way ANOVA. *p* values < 0.05 were considered statistically significant.

## 3. Results

### 3.1. ESA Reduced G2/M Phase Cell Cycle Arrest in the Kidney

**Figure 1:**
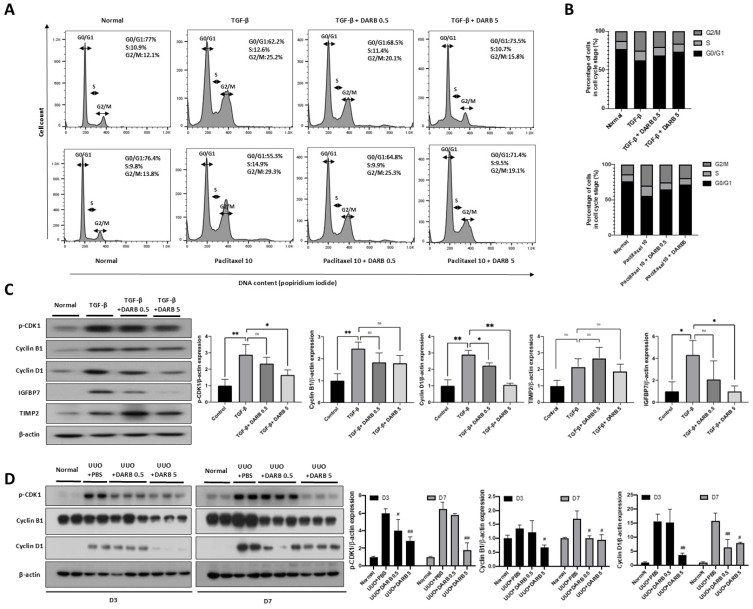
Effect of ESA on cell cycle regulation in the kidney. (**A**) FACS data of TGF-β- or paclitaxel-treated HK-2 cells for 48 h. (**B**) The percentage of HK-2 cells in each phase of the cell cycle (G0/G1, S, and G2/M phases). Both TGF-β- and paclitaxel-treated HK-2 cells showed a significant increase in the proportion of G2/M phase cells, whereas DARB treatment decreased the proportion of G2/M phase cells and promoted the progression of G2/M phase cells to the G0/G1 phase. (**C**) Immunoblot analysis in TGF-β-treated HK-2 cells of G2/M phase-related CDK and cyclin complexes, p-CDK1, Cyclin B1, and Cyclin D1 as well as G1 phase arrest-related markers such as TIMP2 and IGFBP7. (**D**) Immunoblot analysis of p-CDK1, Cyclin B1, and Cyclin D1 shows that DARB treatment significantly reduces the levels of these proteins in the UUO mouse model. Data were expressed as the mean ± SD, with N = 5–6 mice in each group. * *p* < 0.05, ** *p* < 0.001, ns, not significant, # *p* < 0.05, ## *p* < 0.001 vs. UUO + PBS.

To confirm the presence of EPOR in our in vitro model, we first performed Western blot analysis in HK-2 cells. EPOR was clearly detected and showed a dose-dependent increase following DARB treatment, whereas minimal EPOR expression was observed in HeLa cells, which served as a negative control (Supplementary Figure S1). These findings validate the use of HK-2 cells as a suitable model for studying EPOR-mediated effects in renal tubular epithelium. To assess the effect of ESA on cell cycle regulation in the kidney, HK-2 cells were treated with TGF-β or paclitaxel for 48 h, with or without DARB (0.5 and 5 ng/mL) and the cell cycle distribution was analyzed using flow cytometry (Figure 1A,B). TGF-β or paclitaxel treatment significantly increased G2/M phase arrest compared with. However, co-treatment with DARB to TGF-β or paclitaxel treated HK-2 cells reduced G2/M phase arrest and promoted progression to the G0/G1 phase in a dose-dependent manner. Correspondingly, expression of G2/M cell cycle regulatory proteins, including p-CDK1, cyclin B1, and cyclin D1 were altered by treatment of DARB to TGF-β-treated HK-2 cells, showing reduced expression compared with TGF-β treatment alone (Figure 1C). The expression of TIMP2 and IGFBP7, which are associated with G1/S arrest, exhibited an increasing trend in TGF-β-treated HK-2 cells, whereas DARB treatment reduced their expression (Figure 1C). Similar findings were observed in the UUO mouse model, where intraperitoneal DARB administration significantly reduced the levels of G2/M cell cycle regulatory proteins (Figure 1D). These findings suggest that ESA effectively regulates the cell cycle in the kidney, particularly by attenuating G2/M phase arrest and promoting cell cycle progression to the G0/G1 phase.

### 3.2. ESA Exhibited Anti-Fibrotic Activity in the Kidney

**Figure 2:**
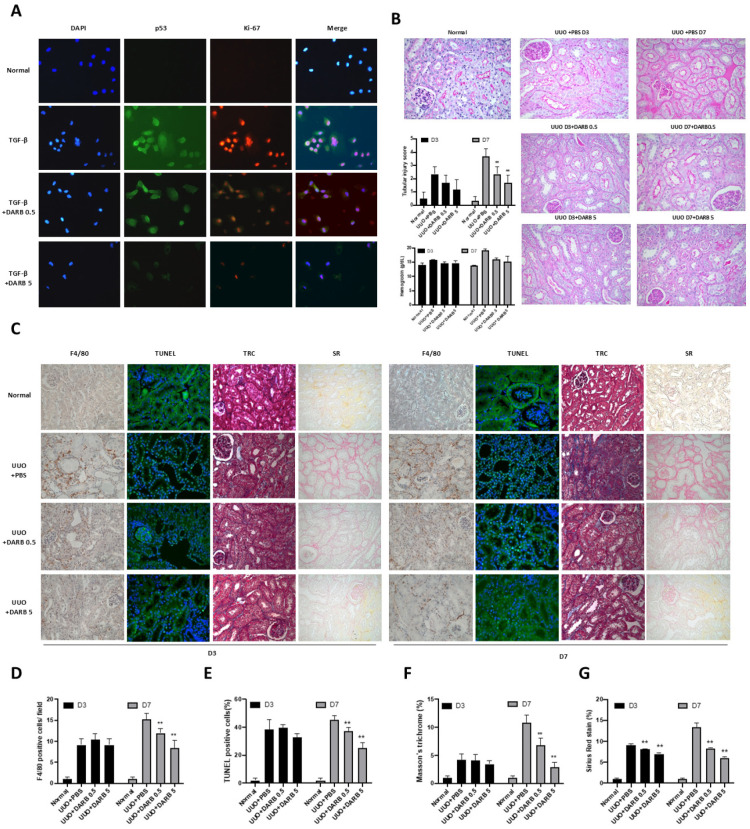
Anti-proliferative activity of ESA in the kidney. (**A**) Co-immunostaining of antibodies against Ki-67 (proliferative cells) and p53 on HK-2 cells stimulated with TGF-β. The density of the staining decreased following high-dose DARB treatment. (**B**) Pathological findings in UUO kidneys after PAS staining. Tubular injury scores based on PAS staining showed that the scores tended to increase over time after UUO and significantly decreased after DARB administration. Hemoglobin levels were measured in UUO mice, both with and without DARB treatment. (**C**) Pathological assessment using Masson’s trichrome (TRC), Sirius red (SR), F4/80, and TUNEL staining. Diffuse tubulointerstitial fibrosis was induced on postoperative days three and seven compared with normal kidney tissue. DARB treatment histologically reversed these fibrotic changes. (**D**) F4/80 staining showed that UUO induced inflammatory cell infiltration; however, DARB treatment attenuated this effect in a dose-dependent manner. (**E**) TUNEL staining was performed to determine the proportion of apoptotic cells after UUO treatment. Apoptotic cells developed after UUO but were reversed after DARB treatment. (**F**) TRC-positive area (%) showed a significant reduction in fibrotic area after DARB administration. (**G**) Quantitative analysis of the SR-positive area (%). Data are expressed as the mean ± SD, with N = 5–6 mice in each group; Magnification ×40; ** *p* < 0.001 vs. UUO + PBS.

To assess the anti-inflammatory and anti-apoptotic effects of ESA during kidney injury, proliferative activity was measured by immunostaining p53 and Ki-67 in TGF-β-treated HK-2 cells, with or without DARB treatment (Figure 2A). In TGF-β-treated HK-2 cells, p53 and Ki-67 expressions were enhanced compared with control, which was significantly attenuated by DARB treatment in a dose-dependent manner. Further histopathological examinations (F4/80 staining and TUNEL assay) in UUO mice models (3 and 7 days) revealed increased inflammatory cell infiltration and apoptotic cells, both which were significantly reversed by DARB. Additionally, Masson’s TRC and SR staining revealed reduced fibrotic changes in UUO mice treated with DARB (Figure 2C–G). PAS staining confirmed the anti-fibrotic effect of ESA in the kidneys, with reduced tubular injury scores following DARB administration. Interestingly, the administration of DARB in UUO mice did not significantly change hemoglobin levels compared with vehicle-treated mice (Figure 2B). These findings suggest that ESA exerts anti-fibrotic effects independent of its hematopoietic effects on the kidneys.

### 3.3. ESA Protects Against Kidney Fibrosis by Attenuating G2/M Cell Cycle Arrest

**Figure 3:**
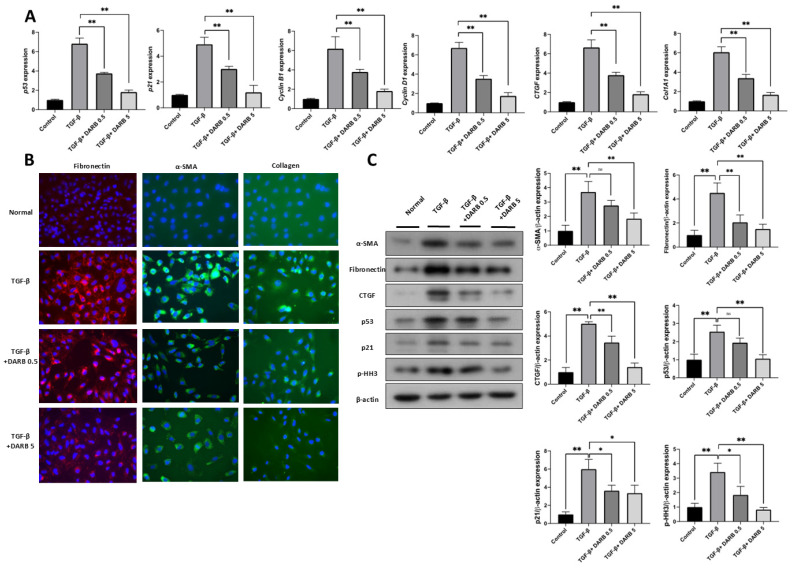
Protective effect of ESA against fibrotic changes in HK-2 cells. (**A**) mRNA expression of *p53, p21, Cyclin B1, Cyclin D1, CTGF, and COL1A1* in HK-2 cells. After TGF-β stimulation, the mRNA expression of profibrotic and G2/M cell cycle-related markers significantly increased; however, DARB dose-dependently reduced these levels. (**B**) Immunofluorescence staining of α-SMA, E-cadherin, and collagen in TGF-β-treated HK-2 cells with or without DARB. (**C**) Immunoblot analysis of profibrotic proteins, including α-SMA, fibronectin, and CTGF as well as G2/M phase-related markers including p53, p21, and p-HH3 in TGF-β-treated HK-2 cells with or without DARB. Data are expressed as mean ± SD; Magnification ×40; * *p* < 0.05, ** *p* < 0.001, ns, not significant.

**Figure 4:**
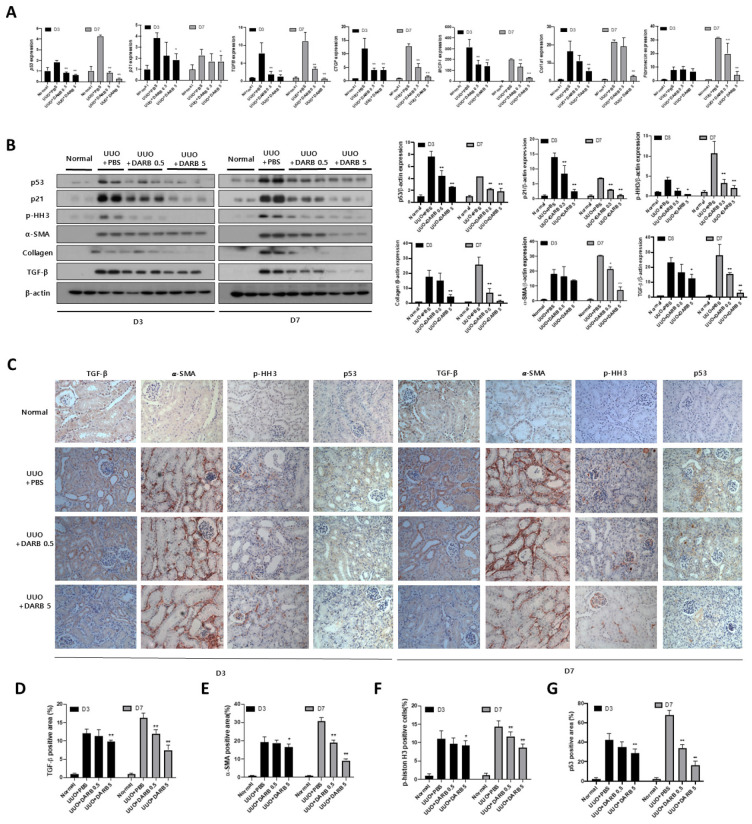
Protective effect of ESA against fibrotic changes in the UUO kidney. (**A**) mRNA expression of *p53* and *p21*, which are associated with G2/M arrest, increased in UUO kidney sections but significantly decreased after DARB treatment. Kidney fibrosis-related markers, including *TGFB*, showed similar results. The levels of pro-inflammatory markers, such as *CTGF* and *MCP-1*, were nearly restored to normal levels following DARB treatment. *COL1A1* and *FN*, the end-products of renal fibrosis, were attenuated by DARB treatment. (**B**) Protein levels of p53 and p21 were reduced by DARB treatment in UUO mouse model. The expression of p–HH3, collagen, and *α*-SMA were significantly decreased. The expression of TGF-β decreased dose-dependently via DARB treatment. (**C**) Immunohistochemical findings of TGF-β, *α*-SMA, p53, and p-Histone H3 staining. Quantitative analysis of (**D**) the TGF-β-positive area (%), (**E**) *α*-SMA-positive area (%), (**F**) p-Histone H3-positive area (%), and (**G**) p53-positive area (%) showed that after UUO, DARB treatment tended to reduce the positive areas of TGF-β, α-SMA, and p53, as well as decreased the percentage of G2/M phase-arrested cells in a dose-dependent manner. Data are expressed at the mean ± SD. N = 5–6 mice in each group; Magnification ×40; * *p* < 0.05, ** *p* < 0.001 vs. UUO + PBS.

To further investigate whether the protective effect of ESA against kidney fibrosis involves G2/M cell cycle regulation, changes in fibrotic and cell cycle-related markers were evaluated during kidney injury with or without DARB treatment. In TGF-β-treated HK-2 cells, the mRNA expressions of fibrotic markers (*CTGF*, and *COL1A1*) (Figure 3A) and G2/M phase-related markers (*p53*, *p21*, *Cyclin B1*, and *Cyclin D1*) were significantly increased; however, DARB treatment reduced these levels (Figure 3A), which was consistent with the observed changes in protein levels (Figure 3C). Furthermore, immunofluorescence staining of α-SMA, E-cadherin, and collagen revealed enhanced expression in TGF-β-treated HK-2 cells, which was significantly attenuated by DARB treatment (Figure 3B). Similar findings were observed in the UUO mice model, where DARB administration reduced the expression of fibrotic markers and p-HH3, a G2/M arrest-related marker (Figure 4A,B). Immunohistochemistry confirmed that DARB treatment significantly reduced the enhanced expression of fibrotic and G2/M arrest markers in UUO mice (Figure 4C–G).

To further clarify whether these anti-fibrotic effects are mediated through EPOR signaling, we treated HK-2 cells with TGF-β and DARB in the presence or absence of an EPOR-blocking peptide. The suppressive effects of DARB on fibrosis-related markers (α-SMA, collagen I, fibronectin) were attenuated by EPOR blockade, supporting that DARB exerts its protective effects, at least in part, via EPOR-dependent mechanisms (Supplementary Figure S2).

### 3.4. ESA Attenuates Kidney Fibrosis by Modulating G2/M Cell Cycle Arrest Through JNK and p38-MAPK Signaling Pathways

**Figure 5:**
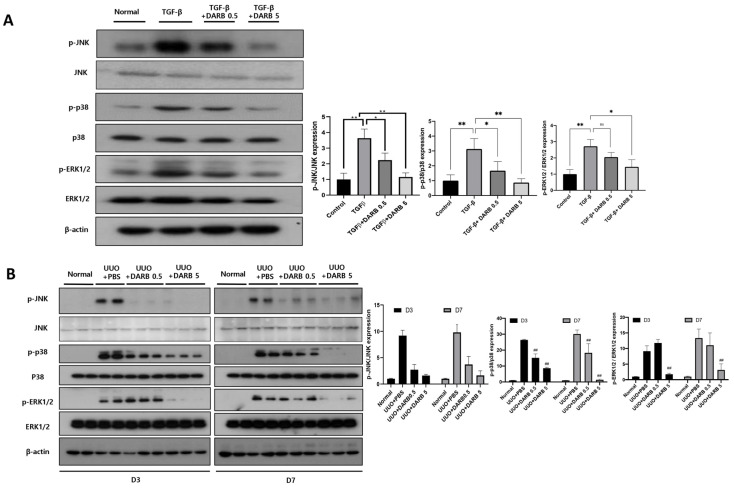
ESA attenuates kidney fibrosis by modulating G2/M cell cycle arrest through JNK and p38-MAPK signaling pathways. (**A**) Immunoblot analysis of p-JNK, p-p38/p38, and p-ERK1/2/ERK1/2 in HK-2 cells. (**B**) Immunoblots of p-JNK, p-p38/p38, and p-ERK1/2/ERK1/2 in the UUO kidney. After the administration of DARB, JNK and p38-MAPK signaling pathways, which contribute to kidney fibrosis, were dose-dependently and significantly suppressed compared with HK-2 cells that were only stimulated with TGF-β and the UUO kidney. Data are expressed as mean ± SD. N = 5–6 mice per group. * *p* < 0.05, ** *p* < 0.001, ns, not significant, ## *p* < 0.001 vs. UUO + PBS.

Given that JNK and p38-MAPK signaling pathways is a key mechanism in kidney fibrosis following kidney injury, we evaluated whether ESA attenuates these pathways during kidney fibrosis via cell cycle modulation. In TGF-β-treated HK-2 cells, the expressions of p-JNK, p-p38/p38, and p-ERK1/2/ERK2 were significantly increased compared with the normal control group, which were reduced by DARB treatment in a dose-dependent manner (Figure 5A). In UUO mice, the expressions of p-JNK, p-p38/p38, and p-ERK1/ERK2 were increased compared with control mice and were significantly suppressed by DARB administration (Figure 5B).

### 3.5. Comparable Effects of ESA and p53 Inhibitors on Cell Cycle Modulation and Kidney Fibrosis

**Figure 6:**
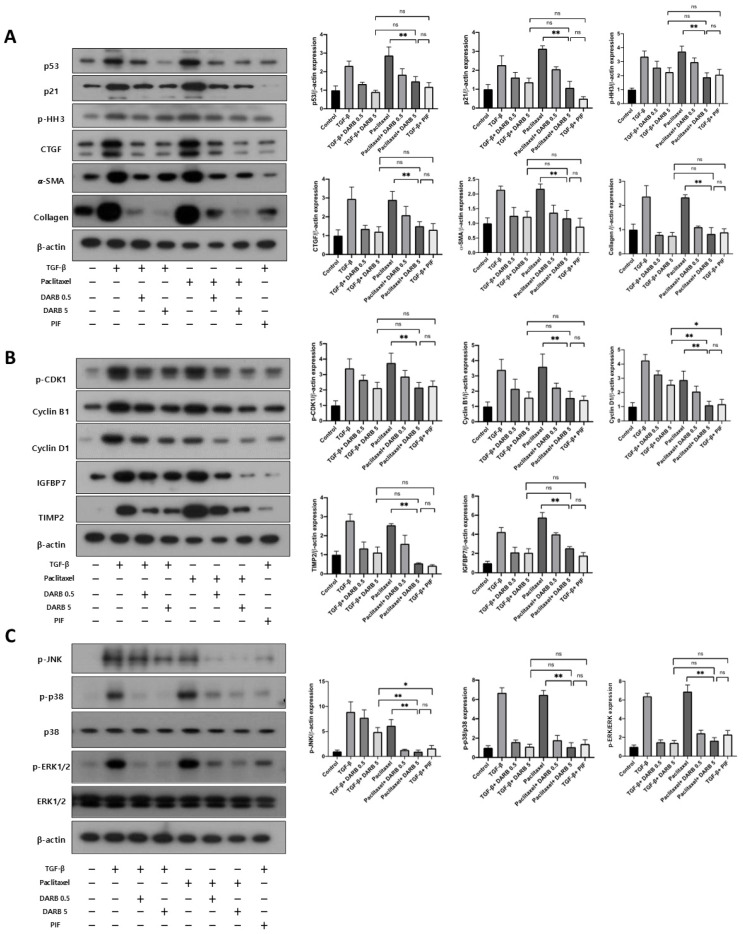
Comparison of anti-fibrotic effects of ESA and the p53 inhibitor. (**A**) In HK-2 cells stimulated with paclitaxel, DARB significantly attenuated the protein expression of p53 and p21. It may be inferred that the number of G2/M-arrested cells was decreased through the p53-p21 pathway. This finding was supported by the protein expression of p-HH3. Similar trends were observed in the expression of CTGF, *α*-SMA, and collagen. (**B**) In HK-2 cells treated with paclitaxel, protein expressions of p-CDK1, Cyclin B1, Cyclin D1, TIMP2, and IGFBP7 showed results similar to those of HK-2 cells treated with TGF-β. DARB administration significantly decreased the expression of these proteins. (**C**) Immunoblot analysis of p-JNK, p-p38/p38, and p-ERK1/2/ERK1/2. After DARB administration, JNK and p38-MAPK signaling pathways, which contribute to kidney fibrosis, were significantly decreased in HK-2 cells stimulated with TGF-β or paclitaxel. Considering these results, the effects of DARB on anti-fibrotic activity, cell regulatory proteins, and the p38-MAPK pathway were comparable to those of a p53 inhibitor (PIF-α). Data are expressed as mean ± SD. * *p* < 0.05; ** *p* < 0.001; ns, not significant.

Finally, we compared the antifibrotic effects of ESA with a p53 inhibitor, an upstream regulator of cell cycle arrest, and a potent antifibrotic agent, in TGF-β or paclitaxel-treated HK-2 cells. The expressions of p53, p21, and p-HH3 were increased in HK-2 cells treated with TGF-β or paclitaxel, whereas treatment with DARB and the p53 inhibitor reduced these levels (Figure 6A). The expressions of fibrotic markers revealed similar changes. Notably, the reduction in the expression of cell cycle arrest and fibrotic markers induced by DARB was comparable to that induced by the p53 inhibitor (Figure 6A,B). Furthermore, both DARB and p53 inhibitor treatments reduced the expressions of the JNK and p38-MAPK signaling pathways, with comparable effects observed between two agents in TGF-β-stimulated HK-2 cells (Figure 6C). Taken together, these results suggest that ESA has an effect comparable to that of a p53 inhibitor in modulating cell cycle arrest and protecting against kidney fibrosis.

## 4. Discussion

ESAs, traditionally used for hematopoietic support, have demonstrated the potential to alleviate kidney fibrosis through their anti-inflammatory, anti-apoptotic, and antifibrotic properties [28]. Studies have shown that ESAs, including DARB, can mitigate tubular injury and interstitial inflammation, thereby enhancing tubular cell survival in various mouse models of nephropathy [29,30]. However, the precise mechanisms underlying their protective effects against kidney fibrosis remain unclear, given the complexity of the associated pathophysiological processes. Kidney fibrosis is driven by multiple mechanisms, one of which involves G2/M cell cycle arrest [31,32,33]. This arrest occurs in tubular epithelial cells after AKI caused by ischemia–reperfusion injury, leading to the abnormal amplification of profibrogenic factors that initiate fibrosis and promote progression to CKD [7,34]. Interventions targeting the reduction in G2/M-arrested cells have been associated with less fibrosis, as demonstrated by pharmacologic approaches such as p53 inhibition and using histone deacetylase inhibitors and mechanical strategies such as unilateral nephrectomy post-ischemia–reperfusion injury [17,35,36,37,38]. Notably, ESAs have also demonstrated non-hematopoietic effects of EPO in erythroblasts, specifically promoting the transition from the G1 to S phase by inhibiting cell cycle regulatory factors such as p27[20]. Moreover, cell cycle regulatory mechanisms include various regulatory molecules such as p53, p21, CDKs, cyclins, and retinoblastoma proteins [39]. Among those, p53 plays a key role in the G2 checkpoint which progresses G2/M transition in response to the damage of proximal tubular cells [40]. In addition, recent studies have identified that uremic toxins, especially indoxyl sulfate (IS), may exacerbate anemia in CKD by disrupting erythropoiesis through EPO-dependent mechanisms, primarily by inducing sub-G1 phase arrest. I have checked and revised all. IS has been shown to induce apoptosis and arrest erythroid development by downregulating key genes such as GATA-1, EPO-R, and β-globin, while also increasing sub-G1 phase accumulation in erythroid progenitor cells. These effects occur even at clinically relevant concentrations and are mediated, at least in part, through EPO-dependent mechanisms. Therefore, IS not only impairs EPO signaling but also interferes with erythroid maturation and survival, compounding the challenge of managing anemia and potentially blunting the beneficial cellular effects of ESAs in CKD. In line with previous studies, our study demonstrated that DARB effectively suppressed the expression of p53 and other cell cycle arrest-related factors, promoting the transition from the G2/M phase in the kidney. These findings suggest that ESAs may exert a protective role in kidney fibrosis by inhibiting cell cycle arrest. In addition, when comparing the antifibrotic effect with that of a p53 inhibitor, DARB showed a similar potency in the kidney, indicating that ESA is a potential therapeutic agent for kidney fibrosis.

Pathways involved in cell cycle progression during the G2/M phase are the ATM and ATR, p38-MAP kinase, and Wee1 kinase signaling pathways [41,42,43]. DNA damage is crucial for activating these pathways, leading to downstream activation of CHK2, which phosphorylates p53 and Cdc25 and inhibits CDK1, resulting in G2/M arrest [41,44,45,46]. However, DNA damage is not the only cause of G2/M arrest. Indeed, many types of stressors, such as ischemic or toxic injury, can also activate the p38-MAPK/MK2 signaling pathway, leading to the subsequent inactivation of Cdc25. MAPK phosphorylates Cdc25, preventing it from activating the CDK1/cyclin A, thereby inhibiting the G2/M transition [47]. Additionally, Wee1 kinase maintains CDK1 in an inactive state through the phosphorylation of its tyrosine residue [48], whereas re-activation of CDK1 occurs through dephosphorylation by Cdc25 [49]. Interestingly, the JNK pathway, one of the major signaling cassettes of MAPK signaling pathway, is activated in G2/M-arrested cells and regulates various cellular events, including the cell cycle, differentiation, survival, apoptosis, and inflammatory responses [7]. Previous studies have suggested that the stress-induced activation of p38 MAPK and JNK signaling is associated with the progression of kidney disease, and blocking this signaling pathway may prevent kidney fibrosis and improve kidney prognosis [50,51]. Borst et al. found sustained JNK signaling pathway activation in proximal tubular cells even one week after severe ischemic injury [52]. This continuous activation of JNK signaling was contributed by G2/M-arrested cells and simultaneous upregulation of profibrotic and inflammatory cytokines [7]. Furthermore, EMT is a crucial process in the pathophysiology of kidney fibrosis, with JNK and p38-MAPK signaling pathways playing significant roles in EMT [51,53,54,55]. Continuous activation of JNK signaling, as observed in proximal tubular cells after severe ischemic injury, results in the upregulation of profibrotic and inflammatory cytokines. In our study, we found that ESA treatment reduced the expression of cell cycle checkpoint markers as well as JNK and p-38 MAPK pathway-related factors along with fibrotic markers such as α-SMA and fibronectin in TGF-β-stimulated HK-2 cells and UUO mice. All these changes resulted in the reduced expression of inflammatory, apoptotic, and fibrotic change markers.

This study has several limitations. First, the anti-inflammatory effects of DARB have not been fully investigated. Although TGF-β plays a critical role in regulating or inducing kidney fibrosis and acts as a potent immunosuppressive cytokine affecting both cell differentiation and proliferation of T-lymphocytes and thymocytes, this study only demonstrated reduced macrophage infiltration and expression of pro-inflammatory markers, including MCP-1 and CTGF. Additional analysis of inflammatory markers, such as TNF-α-, IL-1-, or NF-κB-mediated inflammatory responses, is needed to confirm the causal relationship between DARB use and its anti-inflammatory effects. Second, this study primarily focused on the JNK and p38-MAPK pathways due to their well-established roles in stress-induced cell cycle arrest and fibrosis [7]. However, other mechanisms, such as autophagy regulation, macrophage polarization, and mitochondrial oxidative stress, may also contribute to renal fibrosis and could be modulated by DARB. Further studies are needed to clarify whether DARB influences these alternative pathways as part of its protective effect. Third, our study primarily focused on the early phase of kidney injury, particularly the AKI-to-CKD transition, and demonstrated transient G2/M cell cycle arrest in kidney tubular epithelial cells. We did not observe features consistent with long-term cell cycle arrest or cellular senescence such as senescence-associated secretory phenotypes (SASP) [56]. Therefore, the potential role of cellular senescence and SASP in DARB-mediated anti-fibrotic effects was limited to demonstrate in the present study. Further investigations will be needed to clarify whether modulation of senescence pathways by the long-term DARB treatment contributes to chronic kidney injury. Fourth, we did not perform a full dose-range screening to comprehensively evaluate the dose–response relationship of DARB. Instead, we selected two representative doses based on prior literature and preliminary in vitro findings demonstrating minimal cytotoxicity and sufficient biological activity. Fifth, we did not measure the actual concentrations of DARB in blood or renal tissue following intraperitoneal administration. As a result, we cannot definitively determine whether the renal tubular cells in vivo were exposed to DARB concentrations comparable to those used in vitro. Although the selected in vivo doses were based on previously reported pharmacologically active ranges, future studies incorporating pharmacokinetic analysis will be important to validate tissue-level drug exposure and enhance the translational relevance of our findings. Sixth, our study investigated only one type of ESA, DARB. Given the structural and functional differences between DARB and other ESAs, such as epoetin (α, β) or methoxy polyethylene glycol-epoetin beta, the observed effects should not be generalized to the entire ESA class. Further studies are needed to determine whether similar anti-fibrotic and cell cycle-related effects are shared by other ESAs. Finally, although in vitro and in vivo experiments have consistently demonstrated that DARB inhibits G2/M cell cycle arrest, resulting in an anti-fibrotic effect, these findings should be generalized to human CKD with caution and warrant further investigation.

**Figure 7:**
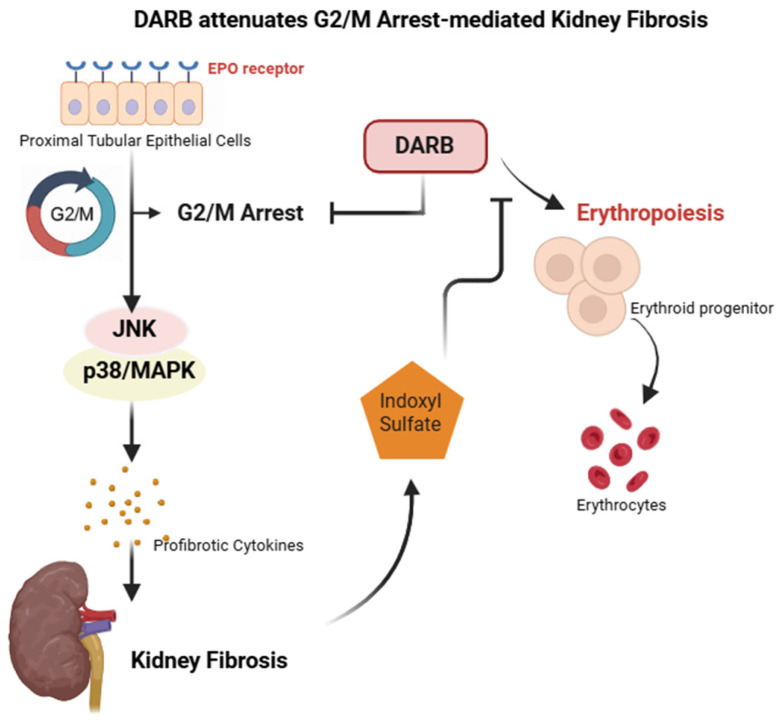
Central illustration DARB significantly reduced kidney fibrosis in TGF-β-stimulated HK-2 cells and UUO mice. This was achieved by rescuing G2/M cell cycle arrest, reduction in inflammation and apoptosis, and antifibrotic effects.

In conclusion, our study demonstrated that DARB significantly reduced kidney fibrosis in TGF-β-stimulated HK-2 cells and UUO mice. This was achieved by rescuing G2/M cell cycle arrest, reduction in inflammation and apoptosis, and antifibrotic effects (Figure 7). These findings highlight the various non-hematopoietic effects of EPO and suggest that ESA is a potential therapeutic option for the treatment of kidney fibrosis.

## Data Availability

The data supporting the findings of the present study are available from the corresponding author upon reasonable request.

## Supplementary Materials

The following supporting information can be downloaded at https://www.mdpi.com/article/10.3390/cells14211662/s1, Supplemental Methods. Antibodies used for Western blotting. Table S1. Primer sequences used for quantitative RT-PCR. Figure S1. EPO receptor expression in HK-2 cells with or without DARB treatment. Figure S2. Effect of EPOR blockade on DARB-mediated anti-fibrotic responses in HK-2 cells.
