# Supplementary material for: Erythropoiesis-Stimulating Agent Protects Against Kidney Fibrosis by Inhibiting G2/M Cell Cycle Arrest
Source: Cells. 2025 Oct 23;14(21):1662. doi: 10.3390/cells14211662 (PMC12609470; doi:10.3390/cells14211662)
Supplement: Supplementary file 1 [file cells-14-01662-s001.zip › cells-3661435-supplementary.pdf]

Online-Only Supplementary Material

Erythropoiesis Stimulating Agent Protects against Kidney Fibrosis by Inhibiting G2/M Cell Cycle Arrest

Donghwan Oh[1†], Jong Hyun Jhee[1†], Soo Hyun Kim[1], Tae Yeon Kim[1], Hoon Young Choi[1,2], and Hyeong Cheon Park[1,2]

[1]Divsion of Nephrology, Department of Internal Medicine, Gangnam Severance Hospital, Yonsei University College of Medicine, Seoul, Republic of Korea

[2]Severance Institute for Vascular and Metabolic Research, Yonsei University College of Medicine, Seoul, Republic of Korea

†These authors contributed equally to this work.

Corresponding Author:

Hyeong Cheon Park, M.D., Ph.D.[1]

[1]Division of Nephrology, Department of Internal Medicine, Gangnam Severance Hospital, Yonsei University College of Medicine, Seoul 06273, Republic of Korea

Phone: 82-02-2019-3306; Fax: 82-02-3463-3882; E-mail: amp97@yuhs.ac

Content list:

Supplemental Methods. Antibodies used in Western blotting

Supplemental Table 1. Primer sequences used for quantitative RT-PCR

Supplemental Figure 1. EPO receptor expression in HK-2 cells with or without DARB treatment

Supplemental Figure 2. Effect of EPOR blockade on DARB-mediated anti-fibrotic responses in HK-2 cells

Supplemental Methods

Antibody information used in Western blotting

Primary antibodies against Erythropoietin receptor (cat. no. ab284292; 1:1000; Abcam, Cambridge, MA, USA), TIMP2 (cat. no. ab180630; 1:5000; Abcam, Cambridge, UK), IGFBP7 (cat. no. ab74169; 1:2000; Abcam, Cambridge, UK), p53 (cat. no. sc-126; 1:1000; Santa Cruz, Dallas, TX, USA), p21 (cat. no. sc-271610; 1:1000; Santa Cruz, Dallas, TX, USA), p-CKD1 (cat. no. ab133463; 1:5000; Abcam, Cambridge, UK), cyclin B1 (cat. no. ab32053; 1:5000; Abcam, Cambridge, UK), cyclin D1 (cat. no. ab134175; 1:5000; Abcam, Cambridge, UK), fibronectin (cat. no. ab2413; 1:1000; Abcam, Cambridge, UK), CTGF (cat.no. ab6992; 1:1000; Abcam, Cambridge, UK), Collagen (cat. no. sc-59772; 1:1000; Santa Cruz, Dallas, TX, USA), TGF-β (cat. no. ab179695; 1:1000; Abcam, Cambridge, UK), p-p38 (cat. no. sc-166182; 1:1000; Santa Cruz, Dallas, TX, USA), p38 (cat. no. ab31828; 1:1000; Abcam, Cambridge, UK), p-ERK1/2 (cat. no. sc-81492; 1:1000; Santa Cruz, Dallas, TX, USA), ERK1/2 (cat. no. 4695; 1:1000; Cell signaling, Danvers, MA, USA), p-JNK (cat. no. 9251; 1:1000; Cell signaling, Danvers, MA, USA), p-HH3 (cat. no. 9701; 1:1000; Cell signaling, Danvers, MA, USA), α-SMA (cat. no.; 1:10000; R&D systems, Minneapolis, MN, USA), and β-actin (cat. no. A5441; 1:10000; Sigma-Aldrich, St Louis, MO, USA) incubated the membranes overnight at 4°C. The membranes were incubated with the corresponding secondary antibodies, including anti-mouse IgG-HRP (cat. no. 7076S; Cell signaling, Danvers, MA, USA) and anti-rabbit IgG-HRP (cat. no. 7074S; Cell signaling, Danvers, MA, USA) for 2 h at 25-30°C temperature.

Supplemental Table S1. Primer sequences for quantitative RT-PCR

Primer			Sequence
Human	*p53*	Forward	5´-GGCCCACTTCACCGTACTAA-3´
		Reverse	5´-GTGGTTTCAAGGCCAGATGT-3´
	p21	Forward	5´-AGTCAGTTCCTTGTGGAGCC-3´
		Reverse	5´-GCATGGGTTCTGACGGACAT-3´
	Cyclin B1	Forward	5´-GACCTGTGTCAGGCTTTCTCTG-3´
		Reverse	5´-GGTATTTTGGTCTGACTGCTTGC-3´
	Cyclin D1	Forward	5´-TCTACACCGACAACTCCATCCG-3´
		Reverse	5´-TCTGGCATTTTGGAGAGGAAGTG-3´
	CTGF	Forward	5´-TGGAAGAGAACATTAAGAAGGGCA-3´
		Reverse	5´-TGCAGCCAGAAAGCTCAAAC-3´
	Col1a1	Forward	5´-GATTCCCTGGACCTAAAGGTGC-3´
		Reverse	5´-AGCCTCTCCATCTTTGCCAGCA-3´
	GAPDH	Forward	5´-TGGCACCCAGCACAATGAA-3´
		Reverse	5´-CTAAGTCATAGTCCGCCTAGAAGCA-3´
Mouse	*p53*	Forward	5´-GTCACAGCACATGACGGAGG-3´
		Reverse	5´-TCTTCCAGATGCTCGGGATAC-3´
	p21	Forward	5´-TCGCTGTCTTGCACTCTGGTGT-3´
		Reverse	5´-CCAATCTGCGCTTGGAGTGATAG-3´
	TGFB	Forward	5´-AAATCAACGGGATCAGCCCC-3´
		Reverse	5´-CGCACACAGCAGTTCTTCTC-3´
	MCP-1	Forward	5´-CACTCACCTGCTGCTACTCA-3´
		Reverse	5´-GCTTGGTGACAAAAACTACAGC-3´
	Col1a1	Forward	5´-GACATGTTCAGCTTTGTGGACCTC-3´
		Reverse	5´-GGGACCCTTAGGCCATTGTGTA-3´
	CTGF	Forward	5´-CCCACACAAGGGCCTCTTC-3´
		Reverse	5´-CCATCTTTGGCAGTGCACAC-3´
	Fibronectin	Forward	5´-ATCATAGTGGAGGCACTGCAGAA-3´
		Reverse	5´-GGTCAAAGCATGAGTCATCTGTAGG-3´
	GAPDH	Forward	5´-TGCATCCTGCACCACCAACT-3´
		Reverse	5´-CCCGTTCAGCTCTGGGATGA-3´

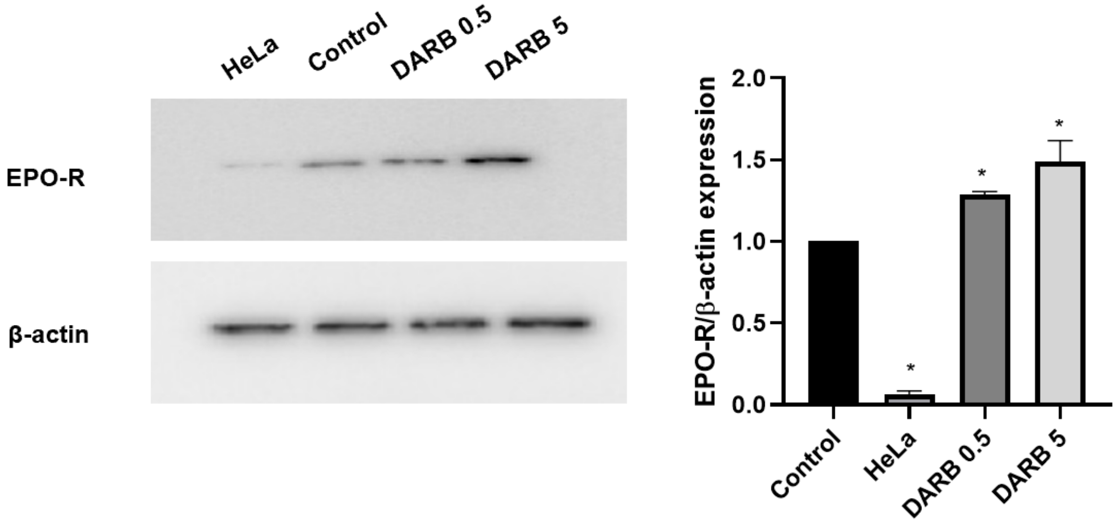

Supplemental Figure S1. EPO receptor expression in HK-2 cells with or without DARB treatment. HK-2 cells and HeLa cells were treated with DARB (0, 0.5, and 5 μg/mL) for 48 hours. EPOR expression was clearly detected in HK-2 cells and showed a dose-dependent increase following DARB treatment, whereas minimal EPOR signal was observed in HeLa cells, which served as a negative control. * $P<0.05$ vs. control

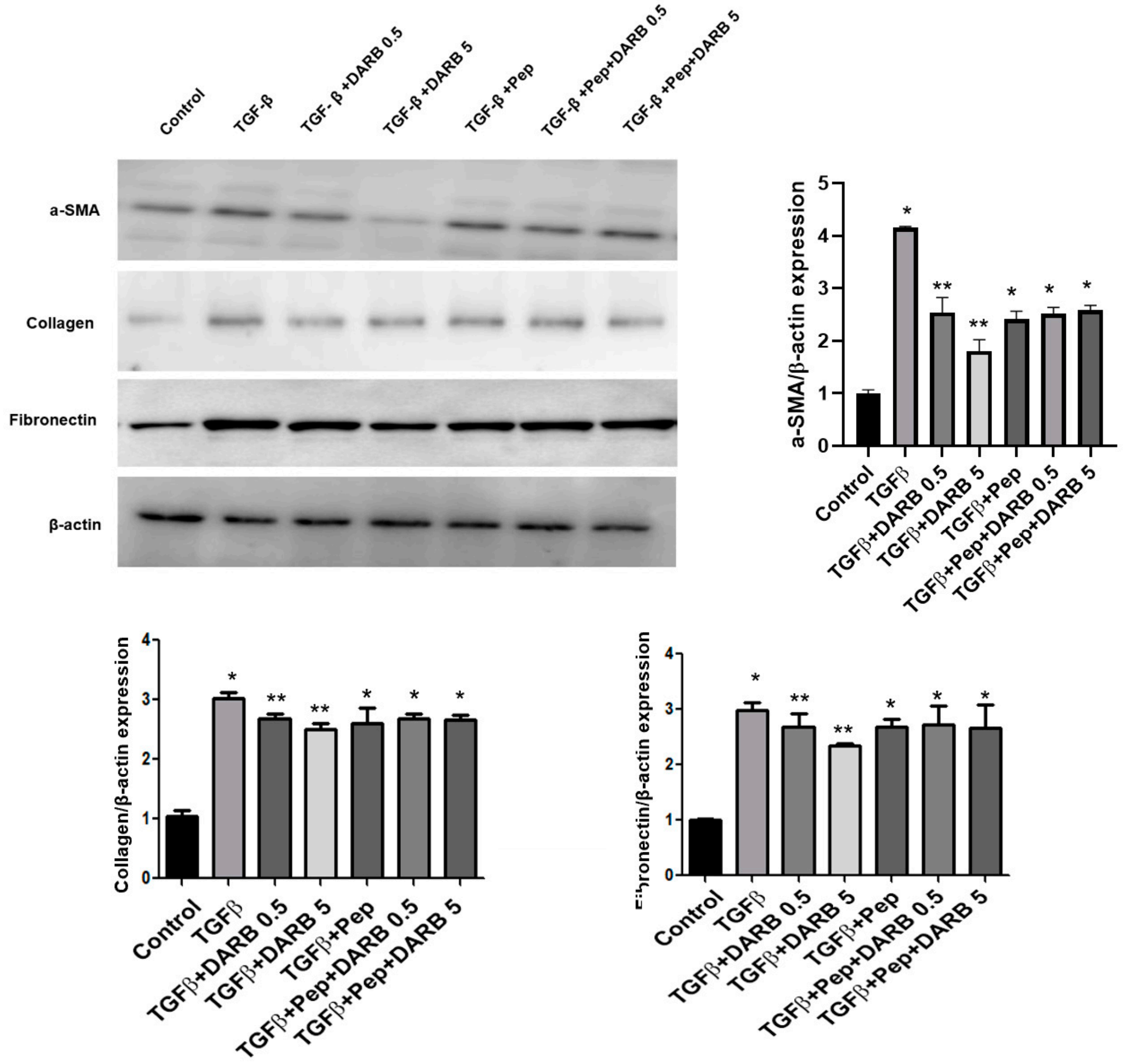

Supplemental Figure S2. Effect of EPOR blockade on DARB-mediated anti-fibrotic responses in HK-2 cells. HK-2 cells were treated with TGF-β (5 ng/mL) with or without DARB (0.5 or 5 μg/mL), in the presence or absence of an EPOR-blocking peptide. The suppressive effects of DARB on fibrosis-related markers (α-SMA, collagen I, fibronectin) were attenuated by EPOR blockade, supporting that DARB exerts its protective effects, at least in part, via EPOR-dependent mechanisms. * *P*<0.05 vs. control, ** *P*<0.05 vs. TGF-β-treated HK-2 cells
